# Plasma oxylipin profile of postpartum dairy cows categorized into different systemic inflammatory grades in the first week after parturition

**DOI:** 10.3168/jdsc.2023-0410

**Published:** 2023-11-18

**Authors:** J.M. Grantz, A. Mukhopadhyay, A.H. Jannasch, C. Ferreira, P.R. Menta, V.S. Machado, R.C. Neves

**Affiliations:** ^1^ Department of Veterinary Clinical Sciences, College of Veterinary Medicine, Purdue University, West Lafayette, IN 47907; ^2^ Metabolite Profiling Facility, Bindley Bioscience Center, Purdue University, West Lafayette, IN 47907; ^3^ Department of Veterinary Sciences, Texas Tech University, Lubbock, TX 79409

## Abstract

Oxygenated polyunsaturated fatty acids (oxylipins) are important mediators of inflammation ranging from pro- to anti-inflammatory actions. Research investigating differences in the oxylipin profile of dairy cows suffering from different degrees of systemic inflammation in the early postpartum period is lacking and can help advance knowledge on potential mechanisms leading to excessive inflammation. The objective of this preliminary study was to evaluate the plasma oxylipin profile of cows classified in 1 of 4 systemic inflammation categories based on plasma haptoglobin (Hp) concentrations assessed on days in milk (DIM) 1, 2, 3, 4, 5, and 7, in addition to the presence or absence of metritis within 10 DIM, and of cows without any clinical diseases within 21 DIM. Groups were classified as follows: (1) cows with a peak Hp concentration ≤3 DIM (EarlyHp) and diagnosed with metritis; (2) cows with a peak Hp concentration 3 < DIM ≤7 (LateHp) and diagnosed with metritis; (3) cows suffering from persistently elevated Hp concentrations assessed on DIM 4 and 7 while remaining apparently healthy during the first 21 DIM (PersistentHp); and (4) apparently healthy cows not suffering from persistently elevated Hp concentrations (LowHp). Six cows from each category were randomly selected from a plasma bank of a parent cohort study including 380 multiparous cows. Plasma samples on DIM 1 and 2, 3 and 4, and 5 and 7 were proportionally pooled to create 3 samples per cow for lipidomic analysis (i.e., pool 1 = DIM 1 and 2; pool 2 = DIM 3 and 4; pool 3: DIM 5 and 7). Statistical analyses were performed using SAS v9.4 (SAS Institute Inc.) and least squares means adjusted for multiple comparisons using the Tukey-Kramer test. Comparisons for EarlyHp and LateHp were only performed on pooled samples from DIM 1 and 2 (i.e., before metritis diagnosis). EarlyHp cows had decreased concentrations of 9(S)-HOTrE compared with LowHp cows of DIM 1–2 pooled samples. LateHp cows had decreased concentrations of 9(10)-DiHOME compared with LowHp cows. Next, we sought to investigate whether cows classified as PersistentHp had time-dependent differences in oxylipin profile versus LowHp cows. PersistentHp cows had decreased concentrations of 19(R)-HETE compared with LowHp cows in a time-dependent manner (only in pooled samples from DIM 5 and 7). Our results identified oxylipins of interest that warrant further investigation to elucidate their in vitro and in vivo functions in the postpartum inflammatory process of dairy cows.

## Highlights

• EarlyHp cows had decreased plasma concentrations of 9(S)-HOTrE.
• LateHp cows had decreased concentrations of 9(10)-DiHOME.
• 9(S)-HOTrE and 9(10)-DiHOME were decreased in PersistentHp compared with LowHp cows.
• PersistentHp cows had decreased plasma concentrations of 19(R)-HETE compared with LowHp cows.
• Oxylipins are involved in dysregulated inflammatory processes in dairy cows.

## Graphical Abstract

**Figure:**
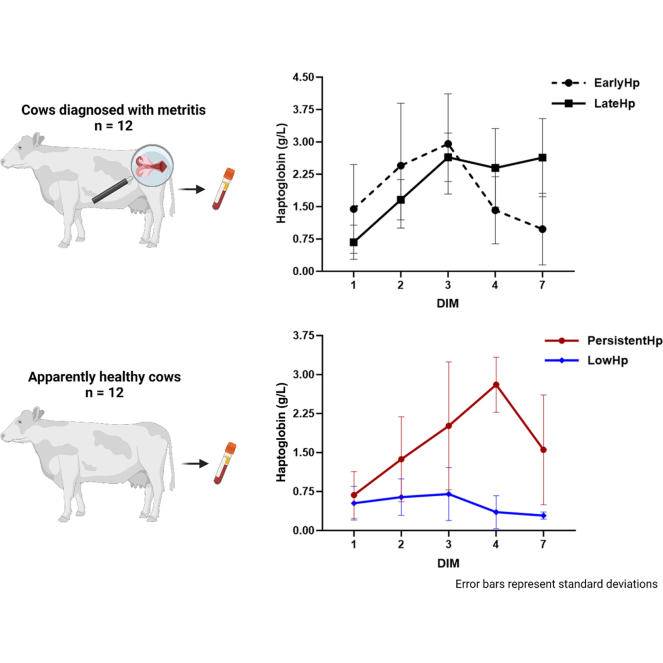
**Summary** Postpartum cows were classified into 1 of 4 systemic inflammation categories based on plasma haptoglobin (Hp) dynamics during the first 7 days in milk (DIM), as follows: (1) peak Hp concentration ≤3 DIM and diagnosed with metritis within 10 DIM (EarlyHp); (2) peak Hp concentration 3 < DIM ≤7 and diagnosed with metritis within 10 DIM (LateHp); (3) cows suffering from persistently elevated Hp concentrations while remaining apparently healthy during the first 21 DIM (PersistentHp); and (4) apparently healthy cows (LowHp). Plasma samples collected on DIM 1 to 5 and 7 were pooled into 3 time points (DIM 1-2, 3-4, and 5, 7) and submitted for lipidomics analysis to compare their oxylipin profile. We detected 32 oxylipins, and our results revealed differences in the oxylipin profile of cows with time-varying differences in peak plasma Hp concentrations. Figure created with BioRender.com.

Inflammation is a physiologically important mechanism to respond to tissue damage and infection. However, excessive or unresolved inflammation has negative impacts on the health and performance of dairy cattle (Bradford et al., 2015). Haptoglobin (**Hp**) is a common marker of systemic inflammation in transition dairy cows and is associated with the degree of immune activation in the early postpartum period (Nightingale et al., 2015; Stevenson et al., 2020). Interestingly, some studies have used Hp concentrations collected in the first week after parturition to discern disorder severity (Huzzey et al., 2009; Sabedra, 2012). Huzzey et al. (2009) demonstrated that cows suffering from severe metritis (cases associated with fever) had a later Hp peak (6 DIM), compared with cows suffering from mild metritis and healthy cows (peak Hp at 3 DIM). Evaluating Hp dynamics in the first week postpartum may be a useful method to classify cows into different degrees of inflammation, and could potentially help understand if different systemic inflammatory responses have any association with known mediators of inflammation.

Enzymatically oxidized lipids represent a broad class of mediators participating in the immune response and inflammation; they are synthesized from PUFA (e.g., arachidonic acid, eicosapentaenoic acid, docosahexaenoic acid) by cyclooxygenase, lipoxygenase, and cytochrome P450. These oxidized lipids are also formed from nonregulated oxidation of PUFA by free radicals (i.e., a response to redox stress), and by nonradical species such as hydrogen peroxide or singlet O_2_ (Smith and Murphy, 2008; Durand et al., 2009). Several oxygenated PUFA, also known as oxylipins or oxylipids, are associated with metabolic syndrome in humans. Studies in dairy cows demonstrated differences in oxylipin profile in the peripartum and their potential use as a disorder predictor (Raphael et al., 2014; Putman et al., 2022). To the best of our knowledge no studies have used Hp dynamics in the first week after parturition to categorize cows into different patterns of systemic inflammation to evaluate differences in their oxylipin profile. Studies identifying lipid mediators that are associated with the degree of the systemic inflammatory response in the postpartum can help with more targeted studies and in-depth analysis that can enhance cow health.

Our objective was to perform a preliminary study to evaluate the plasma oxylipin profile of cows classified in one of 4 systemic inflammation categories based on plasma Hp dynamics assessed on DIM 1, 2, 3, 4, 5, and 7, in addition to the presence or absence of metritis within 10 DIM, and of cows without any clinical diseases within 21 DIM. The study was designed to detect at least a 2.0 ng/mL difference in plasma oxylipins (SD: 1.2 ng/mL) between any of the 4 systemic inflammation group categories for 80% power and α = 0.05. A total of 6 cows per group (n = 24) were needed. Our sample size also follows recommendation by the Purdue University Metabolite Profiling Facility for discovery experiments where at least 4 biological replicates are needed. We hypothesized that cows classified as having increased systemic inflammation based on time-varying Hp peaks would have decreased anti-inflammatory and increased pro-inflammatory oxylipins and time-dependent associations compared with healthy cows.

The cows used in this study were from a parent study (Menta et al., 2021), and animal procedures were approved by the Texas Tech University Institutional Animal Care and Use Committee (protocol 18035–04); additional details on the farm and cow cohort are described in the article. Briefly, blood samples were obtained from multiparous cows on DIM 1, 2, 3, 4, 5, and 7, immediately cooled down in thermo-regulating modules, and plasma stored in a −80°C freezer. Additionally, Metricheck scores were assessed on DIM 4, 7, and 10 to determine a metritis diagnosis. For this study, cows were classified into 1 of 4 inflammation categories based on plasma Hp dynamics during the first 7 DIM and in the presence or absence of a metritis diagnosis within 10 DIM. The groups were as follows: (1) cows with a peak Hp concentration ≤3 DIM (**EarlyHp**) and diagnosed with metritis; (2) cows with a peak Hp concentration 3 < DIM ≤7 (**LateHp**) and diagnosed with metritis; (3) cows suffering from persistently elevated Hp concentrations assessed on DIM 4 and 7 while remaining free from any clinical disease during the first 21 DIM (**PersistentHp**; Martins et al., 2021); and (4) cows not suffering from persistently elevated Hp concentrations that were free from any clinical disease during the first 21 DIM (**LowHp**). Of note, metritic cows had no other comorbidities or risk factors known to increase metritis incidence (i.e., dystocia, retained placenta, milk fever). Six cows from each group (experimental units) were randomly selected using a web-based random integer generator (https://www.random.org/) from 4 lists (each list representing a cow group) with cow IDs organized in ascending order. At the time of lipid extraction, samples were proportionally pooled for further lipidomic analysis and performed at the Metabolite Profiling Facility, Bindley Bioscience Center, Purdue University.

All reagents used for plasma lipid extraction were HPLC grade. Before sample analyses, protocol optimization was performed to maximize analytical coverage of the targets of interest. Briefly, 500 µL of the pooled plasma samples were mixed with 1.5 mL of methanol with 0.1% formic acid, and 10 µL of an internal standard mixture containing the oxylipins of interest (Cayman Chemical). Samples were vortexed for 10 s and centrifuged at 16,000 × *g* for 10 min, 4°C. Supernatants were transferred to a microtube and evaporated in a SpeedVac Savant SPD2030 (Thermo Fisher Scientific Inc.). Dried extracts were reconstituted with 50 µL of a 1:1 ratio of methanol/ultrapure water and then transferred to an autosampler vial for immediate analysis in an Agilent 1290 Infinity II liquid chromatography system (**LC**) coupled to an Agilent 6470 Triple Quadrupole mass spectrometer (MS/MS; Agilent Technologies Inc.). An Acquity UPLC BEH C18 2.1 mm × 100 mm, 1.7 µm column was used for LC separation (Waters Corporation). Mobile phase solvents were 0.1% formic acid in water (buffer A) and 0.1% formic acid in acetonitrile (buffer B), with gradient conditions as follows: time 0 and 0.5 min (90% A, 10% B); time 15 and 16 min (100% B); time 16.1 and 18 min (90% A, 10% B). The flow rate was 0.4 mL per min and the column heated to 40°C. Multiple reaction monitoring was used for MS analysis according to Table 1. Data were acquired in negative electrospray ionization mode. The jet stream electrospray ionization interface had a gas temperature of 325°C, gas flow rate of 7 L/min, nebulizer pressure of 310 kPa, sheath gas temperature of 250°C, sheath gas flow rate of 7 L/min, capillary voltage of 3,500 V in negative mode, and nozzle voltage of 500 V. The ΔEMV voltage was 400 V. Data were processed with the Agilent MassHunter Quantitative Analysis software version 10.1.

**Table 1:** Abbreviation, multiple reaction monitoring (MRM) quantifier, and PubChem compound identifier of 32 plasma oxylipins detected in the plasma of postpartum dairy cows categorized into different grades of systemic inflammation

Abbreviation	Oxylipin	MRM	PubChem compound identifier
DHA metabolite			
DHA	Docosahexaenoic acid	327.4 → 283.4	445,580
7(8)-EpDPA	(±)-(4Z)-6-[3-(2Z,5Z,8Z,11Z)-2,5,8,11-Tetradecatetraen-1-yl-2-oxiranyl]-4-hexenoic acid	343.4 → 112.8	89,861,878
7(8)-DiHDPA	(±)7,8-Dihydroxydocosa-4Z,10Z,13Z,16Z,19Z-pentaenoic acid	361.4 → 127.2	16,061,144
10(11)-EpDPA	(±)-(4Z,7Z)-9-[3-(2Z,5Z,8Z)-2,5,8-undecatrien-1-yl-2-oxiranyl]-4,7-nonadienoic acid	343.4 → 153.2	124,403,576
10(11)-DiHDPA	(±)10,11-Dihydroxy-4Z,7Z,13Z,16Z,19Z-docosapentaenoic acid	361.4 → 153.2	16,061,145
13(14)-EpDPA	(±)13,14-Epoxy docosapentaenoic acid	343.4 → 161.3	124,407,158
19(20)-EpDPA	(±)-(4Z,7Z,10Z)-12-[3-(2Z,5Z)-2,5-octadien-1-yl-2-oxiranyl]-4,7,10-dodecatrienoic acid	343.4 → 241.6	11,631,565
19(20)-DiHDPA	(±)19,20-Dihydroxy-4Z,7Z,10Z,13Z,16Z-docosapentaenoic acid	361.5 → 229.4	16,061,148
14(S)-HDHA	14S-Hydroxy-4Z,7Z,10Z,12E,16Z,19Z-docosahexaenoic acid	343.5 → 281.5	52,921,998
Resolvin D1	7S,8R,17S-Trihydroxy-4Z,9E,11E,13Z,15E,19Z-docosahexaenoic acid	375.4 → 141.4	44,251,266
Protectin D1	10R,17S-Dihydroxy-4Z,7Z,11E,13E,15Z,19Z-docosahexaenoic acid	359.4 → 153.4	16,042,541
Maresin 1	7R,14S-Dihydroxy-4Z,8E,10E,12Z,16Z,19Z-docosahexaenoic acid	359.4 → 341.4	60,201,795
EPA metabolite			
EPA	Eicosapentaenoic acid	301.4 → 257.4	5,282,847
5(6)-DiHET	(±)5,6-Dihydroxy-8Z,11Z,14Z-eicosatrienoic acid	337.4 → 145.2	5,283,142
8(9)-EpETE	(±)8,9-Epoxy-5Z,11Z,14Z,17Z-eicosatetraenoic acid	317.4 → 155.2	16,061,086
8(9)-DiHET	(±)8,9-Dihydroxy-5Z,11Z,14Z-eicosatrienoic acid	337.4 → 185.2	5,283,144
11(12)-EpETE	(±)11,12-Epoxy-5Z,8Z,14Z,17Z-eicosatetraenoic acid	317.4 → 195.2	16,061,087
11(12)-DiHET	(±)11,12-Dihydroxy-5Z,8Z,14Z-eicosatrienoic acid	336.5 → 167.2	5,283,146
14(15)-DiHETE	(±)14,15-Dihydroxy-5Z,8Z,11Z,17Z-eicosatetraenoic acid	337.4 → 207.3	16,061,119
17(18)-DiHETE	(±)17,18-Dihydroxy-5Z,8Z,11Z,14Z-eicosatetraenoic acid	336.5 → 203.3	16,061,120
18-HEPE	(±)-18-Hydroxy-5Z,8Z,11Z,14Z,16E-eicosapentaenoic acid	317.5 → 299.4	16,061,132
ARA metabolite			
ARA	Arachidonic acid	303.4 → 259.4	444,899
11(12)-EET	(±)11,(12)-Epoxy-5Z,8Z,14Z-eicosatrienoic acid	319.4 → 167.2	5,353,269
14(15)-EET	(±)14(15)-Epoxy-5Z,8Z,11Z-eicosatrienoic acid	319.4 → 219.3	5,283,205
15(S)-HETE	15S-Hydroxy-5Z,8Z,11Z,13E-eicosatetraenoic acid	319.6 → 219.6	5,280,724
19(R)-HETE	19R-Hydroxy-5Z,8Z,11Z,14Z-eicosatetraenoic acid	319.4 → 231.2	11,244,126
20-HETE	20-Hydroxy-5Z,8Z,11Z,14Z-eicosatetraenoic acid	319.4 → 245.2	5,283,157
Leukotriene B_4_	5S,12R-Dihydroxy-6Z,8E,10E,14Z-eicosatetraenoic acid	335.4 → 317.4	5,280,492
PGE_2_	9-Oxo-11α,15S-dihydroxy-prosta-5Z,13E-dien-1-oic acid	351.4 → 333.4	5,280,360
Linoleic acid metabolite			
9(10)-DiHOME	(±)9,10-Dihydroxy-12Z-octadecenoic acid	313.4 → 297.4	25,320,858
α-Linolenic acid metabolite			
9(S)-HOTrE	9S-Hydroxy-10E,12Z,15Z-octadecatrienoic acid	293.3 → 275.4	6,439,873
γ-Linolenic metabolite			
13(S)-HOTrE(γ)	13S-Hydroxy-6Z,9Z,11E-octadecatrienoic acid	293.3 → 275.4	5,282,971

Statistical analyses were performed in SAS v9.4 (SAS Institute Inc.). Oxylipin distributions were initially assessed for normality via the UNIVARIATE procedure by visual assessments of quantile-quantile (**Q-Q**) plots and the use of Shapiro-Wilk tests. Comparisons of EarlyHp and LateHp were only performed on pooled samples from DIM 1 and 2 to best ensure the likelihood of modeling a risk factor (i.e., reduced probability of changes in oxylipins being a consequence of metritis being fully established). Analysis of variance to compare group means between EarlyHp, LateHp, PersistentHp, and LowHp for pooled DIM 1 and 2 samples was assessed with the MIXED procedure. Repeated measures models were built to assess longitudinal differences in oxylipin concentrations between PersistentHp and LowHp cows (time point 1 = pooled DIM 1 and 2 samples, time point 2 = pooled DIM 3 and 4 samples, and time point 3 = DIM 5 and 7 samples) with the MIXED procedure. The first-order autoregressive structure with homogeneous variance was used to account for the association of residuals from the same experimental unit (i.e., cow) and assessed to yield the best model fit (lowest Akaike information criterion). In all models, homoscedasticity was assessed by visual inspection of model residuals via Q-Q plots, and potential differences in oxylipin concentrations between groups and time (repeated measures) were controlled for familywise error rate using the Tukey-Kramer test.

We targeted the identification of 48 oxylipins and 32 had detectable concentrations (Table 1). Our targets included oxylipins originating from the cyclooxygenase, lipoxygenase, and cytochrome P450 pathways from arachidonic acid, eicosapentaenoic acid, docosahexaenoic acid, α- and γ-linolenic acid and known to be involved in inflammatory processes. Moreover, all targets assessed needed to have internal standards available as a prerequisite. Of note, some parent compounds were not targeted due to their metabolically unstable nature (rapid metabolism in vivo) or the lack of availability of internal standards. Results of the analysis from DIM 1–2 pooled plasma samples are shown in Table 2. EarlyHp cows had decreased plasma concentrations of 9(S)-HOTrE in pooled samples from DIM 1–2 compared with LowHp cows (0.96 and 4.80 ± 0.93 ng/mL, respectively; *P* = 0.04). An oxylipin derived from the action of 5-lipoxygenase and cytochrome P450 on α-linolenic acid, 9(S)-HOTrE is an agonist of the peroxisome proliferator-activated receptor α (PPARα) (van der Krieken et al., 2018; Heintz et al., 2022). In murine models of systemic inflammation, for instance, PPARα exerts anti-inflammatory effects by suppressing IL-6 expression and the acute-phase response (Gervois et al., 2004; Han et al., 2006). To the best of our knowledge, no other studies have identified 9(S)-HOTrE in the plasma of transition dairy cows, and our results shed light on an important metabolite that may contribute to cows suffering from excessive systemic inflammation in the postpartum. More studies are needed to identify the role of 9(S)-HOTrE in dairy cows. No other significant differences were observed for cows categorized as EarlyHp compared with LateHp, PersistentHp, or LowHp cows on DIM 1–2 pooled samples.

**Table 2:** Least squares means for oxylipins in pooled plasma samples from DIM 1 to 2 in cows categorized into different inflammatory categories and group effect (*P*-value)^1^

Variable	EarlyHp	LateHp	PersistentHp	LowHp	SEM	Group
DHA	3,755.48	4,002.25	3,237.38	2,357.65	722.4	0.43
7(8)-EpDPA	0.93	1.14	0.64	0.61	0.22	0.31
7(8)-DiHDPA	0.73	0.75	0.85	0.79	0.11	0.88
10(11)-EpDPA	0.18	0.16	0.13	0.21	0.04	0.57
10(11)-DiHDPA	0.24	0.23	0.24	0.20	0.05	0.92
13(14)-EpDPA	0.03	0.03	0.03	0.03	0.01	0.99
19(20)-EpDPA	0.73	0.61	0.60	0.48	0.20	0.85
19(20)-DiHDPA	0.35	0.53	0.30	0.30	0.10	0.33
14(S)-HDHA	0.24	0.19	0.14	0.23	0.09	0.88
Resolvin D1	0.21	0.20	0.18	0.17	0.02	0.39
Protectin D1	0.01	0.007	0.01	0.006	0.00	0.24
Maresin 1	0.33	0.38	0.33	0.25	0.07	0.68
EPA	86.37	89.93	91.10	70.19	14.43	0.72
5(6)-DiHET	0.10	0.08	0.09	0.06	0.03	0.83
8(9)-EpETE	1.04	0.57	0.42	0.89	0.20	0.16
8(9)-DiHET	0.17	0.17	0.14	0.10	0.03	0.33
11(12)-EpETE	0.90	0.75	0.96	1.35	0.17	0.10
11(12)-DiHET	0.29	0.35	0.34	0.25	0.05	0.52
14(15)-DiHETE	0.35	0.41	0.36	0.27	0.06	0.48
17(18)-DiHETE	15.11	13.61	13.61	13.52	0.73	0.37
18-HEPE	22.28	21.83	21.35	18.72	3.75	0.91
ARA	1,746.16	1,623.45	1,668.77	1,276.07	269.83	0.63
11(12)-EET	0.72	0.31	0.48	0.04	0.26	0.34
14(15)-EET	0.08	0.01	0.04	0.04	0.03	0.64
15(S)-HETE	3.30	3.00	3.00	3.00	0.15	0.41
19(R)-HETE	0.30	0.31	0.31	0.20	0.07	0.66
20-HETE	0.83	0.61	0.49	0.50	0.16	0.43
Leukotriene B_4_	0.21	0.19	0.19	0.19	0.01	0.41
PGE_2_	0.24	0.21	0.26	0.20	0.02	0.24
9(10)-DiHOME	1.54^ab^	1.07^b^	1.25^b^	2.43^a^	0.27	0.01
9(S)-HOTrE	0.96^b^	1.52^ab^	1.80^ab^	4.80^a^	0.93	0.04
13(S)-HOTrE(γ)	0.91	1.92	1.82	3.76	0.97	0.24

a,b Different superscripts within a row denote significance at *P* ≤ 0.05.

1 Concentrations are presented in nanograms per milliliter with their associated SEM.

Cows categorized as LateHp had decreased concentrations of 9(10)-DiHOME compared with LowHp cows (1.07 and 2.43 ± 0.27 ng/mL, respectively; *P* = 0.01) in pooled samples from DIM 1–2. An oxylipin originated from the metabolism of linoleic acid by cytochrome P450, 9(10)-DiHOME is considered a leukotoxin diol, and when methylated, has been shown to be involved in the inhibition of neutrophil oxidative burst in HL-60 cell lines (Sisemore et al., 2001; Newman et al., 2005; Thompson and Hammock, 2007; Hildreth et al., 2020). Interestingly, this is the second study demonstrating that transition cows with decreased concentrations of DiHOME have an increased incidence of postpartum disorders such as retained placenta and metritis (Putman et al., 2022). Polymorphonuclear leukocytes from cows that develop retained placenta and metritis have decreased phagocytic capacity (Kimura et al., 2002; Chebel, 2021). Therefore, the decreased concentrations of 9(10)-DiHOME in the plasma of LateHp cows are suggestive of an impaired innate immune response compared with LowHp cows. No other significant differences were found in the comparison of oxylipin profiles of LateHp cows against EarlyHp, PersistentHp, and LowHp cows.

The small number of differences in the oxylipin profile of cows in the EarlyHp and LateHp groups compared with LowHp cows were unexpected and could be a result of our small sample size. Future studies with a greater number of cows are needed to better evaluate oxylipin profiles in cows with time-varying peak Hp concentrations.

Our group recently demonstrated that a subpopulation of apparently healthy cows suffers from a more prolonged state of systemic inflammation in the first week after parturition (Martins et al., 2021). Despite that, it is unknown what mechanisms lead to a more persistent systemic inflammation state in the early postpartum. Therefore, we performed time-series analyses to evaluate which oxylipins were associated with a more prolonged inflammatory state and to gain more biological insight. PersistentHp cows had an overall increased plasma concentration of prostaglandin E_2_ (**PGE_2_**), a cyclooxygenase-derived oxylipin, compared with LowHp cows (0.26 vs. 0.20 ± 0.01 ng/mL, respectively; *P* = 0.006), with no time effect (*P* = 0.95), or group by time point interaction (*P* = 0.96). Whether the overall difference in PGE_2_ concentrations is of biological relevance remains to be elucidated. However, differences of approximately 50 pg/mL (0.05 ng/mL) are encountered in tissues under a chronic inflammatory process, and in vitro studies involving PGE_2_ manipulations reinforce the potent actions of this metabolite in very low concentrations (Fournier et al., 1997; Tchetina et al., 2007). Prostaglandin E_2_ is a well-studied oxylipin that is known to have pro-inflammatory properties and is documented as a mediator of inflammation in several models. Moreover, PGE_2_ has been studied in dairy cattle where it was identified as a pro-inflammatory oxylipin that differs significantly between diseased and apparently healthy cows (Raphael et al., 2014; Sordillo, 2018; Putman et al., 2022). Prostaglandin E_2_ can produce a pro-inflammatory response in human and mouse models by acting as a vasodilator to allow for the influx of immune cells to injured tissues (Agard et al., 2013). Additionally, a study utilizing human primary cells has shown that PGE_2_ promotes the activation of CD4^+^ T-helper cells, which induce the production of the pro-inflammatory cytokine, interleukin 17 (Napolitani et al., 2009). Altogether, the classification of cows into a PersistentHp category is substantiated by increased plasma concentrations of PGE_2_ compared with LowHp cows.

PersistentHp cows had decreased plasma concentration of 9(10)-DiHOME compared with LowHp cows (1.52 vs. 2.62 ± 0.17 ng/mL, respectively; *P* < 0.001), with no time effect (*P* = 0.44) or group by time point interaction (*P* = 0.97). Also, PersistentHp cows had a decreased plasma concentration of 9(S)-HOTrE compared with LowHp cows (2.64 vs. 6.0 ± 0.68 ng/mL; *P* = 0.001), with no time effect (*P* = 0.24) or group by time point interaction (*P* = 0.51). PersistentHp cows likely had an impaired innate immune response as 9(10)-DiHOME is a potent inhibitor of neutrophil oxidative burst, and can minimize collateral tissue damage (Thompson and Hammock, 2007). Moreover, decreased concentrations of 9(S)-HOTrE in PersistentHp cows demonstrate that those cows likely had an impaired anti-inflammatory capacity.

PersistentHp cows had an overall decreased plasma concentration of 15(S)-HETE compared with LowHp cows (3.000 vs. 3.004 ng/mL ± 0.00, respectively; *P* = 0.003), with no time effect (*P* = 0.22) or group by time point interaction (*P* = 0.33). Production of 15(S)-HETE results from the oxidation of arachidonic acid by lipoxygenases. This oxylipin has been documented to promote inflammation by disrupting endothelial tight junctions in murine models and, in humans, has been shown to inhibit neutrophil migration (Takata et al., 1994; Chattopadhyay et al., 2014). However, it is important to note that despite the observed significant difference for this oxylipin, it is unlikely to be biologically meaningful due to the magnitude of the difference found.

A significant group by time point interaction was present for plasma concentrations of 19(R)-HETE between PersistentHp and LowHp cows (*P* = 0.01). After a Tukey-Kramer adjustment for multiple comparisons, 19(R)-HETE concentrations in pooled samples from DIM 5 and 7 were significantly greater in LowHp cows compared with PersistentHp (Figure 1A). This oxylipin is formed from the oxidation of arachidonic acid via cytochrome P450. Similar to 15(S)-HETE, 19(R)-HETE has not been heavily studied for its role in inflammation. However, it has primarily been studied for its role in regulating cardiovascular, renal, and pulmonary functions (Tunaru et al., 2016). Pretreatment of murine platelets with 19(S)-HETE prevented thrombin-induced platelet activation and aggregation, thereby highlighting a function of this oxylipin that could be of relevance to the inflammatory response. Recent literature suggests the function of activated platelets extends beyond hemostasis and is a key cell mediating inflammation (Coppinger et al., 2007; Thomas and Storey, 2015). Of note, platelet α-granules contain molecules responsible for hemostasis, as well as inflammatory cytokines and chemokines (Coppinger et al., 2007; Semple et al., 2011; Thomas and Storey, 2015). Upon activation, platelets will release the contents of α-granules which contain numerous cytokines that predominantly help regulate the migration of leukocytes to sites of injury or inflammation, promote ROS production, and aid in phagocytosis of other cells (Thomas and Storey, 2015). Therefore, the ability of 19(S)-HETE to prevent platelet activation is an area of interest, and future studies are needed to determine if this oxylipin is capable of mediating inflammation via platelet inhibition in dairy cattle.

**Figure 1:**
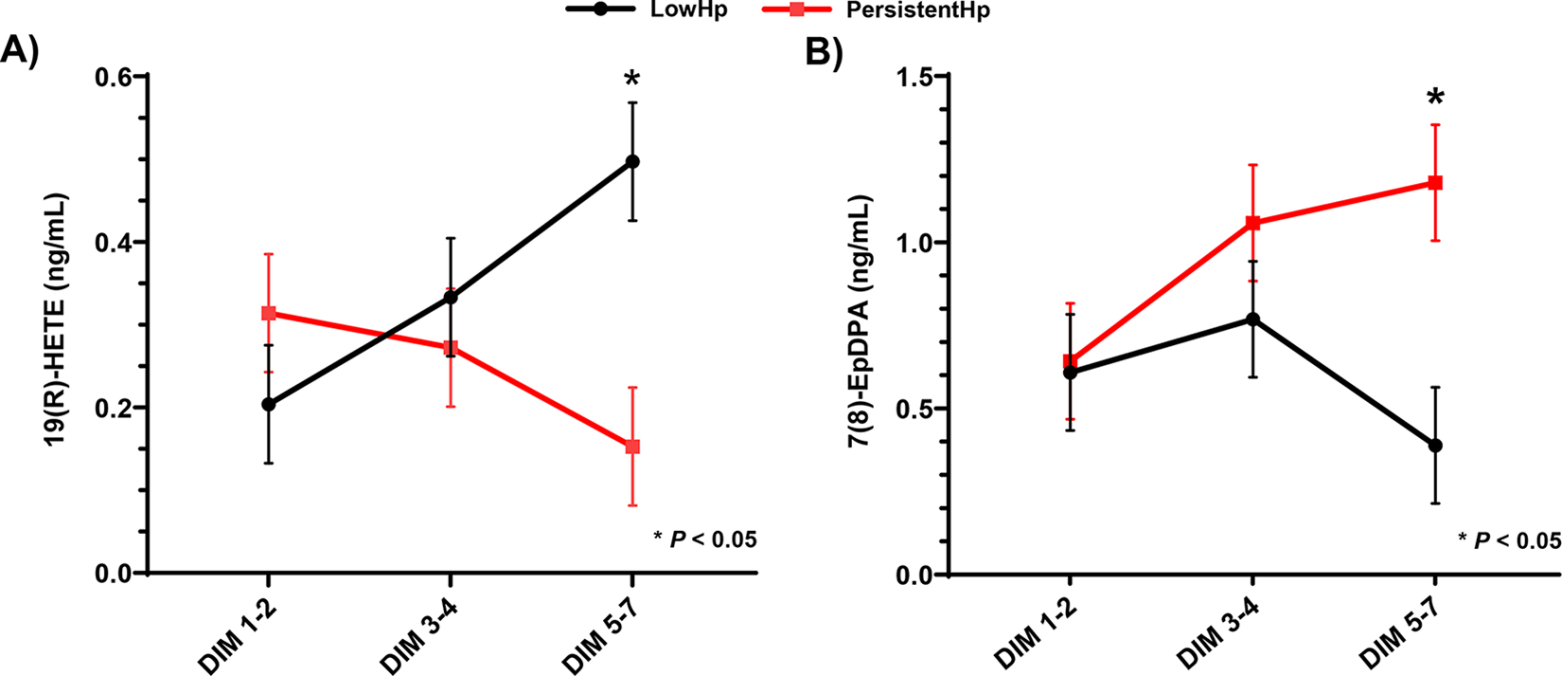
Concentrations of 19(R)-HETE (A) and 7(8)-EpDPA (B) on pooled plasma samples from PersistentHp and LowHp cows on DIM 1–2, 3–4, and 5–7. A group by time point interaction (*P* = 0.01) was present for 19(R)-HETE and a tendency (*P* = 0.10) for 7(8)-EpDPA. After Tukey-Kramer adjustment for multiple comparisons, pooled samples from DIM 5 and 7 were significantly different (*P* < 0.05) between PersistentHp and LowHp. PersistentHp = cows suffering from persistently elevated Hp concentrations assessed on DIM 4 and 7 while remaining free from any clinical disease during the first 21 DIM; LowHp = cows not suffering from persistently elevated Hp concentrations that were free from any clinical disease during the first 21 DIM. Error bars represent SEM.

There was a tendency for a group by time point interaction for 7(8)-EpDPA between the PersistentHp and Low Hp cows (*P* = 0.10; Figure 1B). After adjustment for multiple comparisons, PersistentHp cows had increased concentrations of 7(8)-EpDPA compared with LowHp cows on pooled samples from DIM 5 and 7. This oxylipin is derived from the oxidation of DHA via cytochrome P450, and has been documented in humans to increase the nuclear factor kappa-light-chain-enhancer of activated B cells (NF-κB) gene expression (Borsini et al., 2021). Increased expression and activity of NF-κB are known to have pro-inflammatory effects by increasing the gene expression of pro-inflammatory cytokines. Therefore, the tendency of PersistentHp cows to have increased plasma concentrations of 7(8)-EpDPA could be a factor contributing to their consistently elevated concentration of Hp compared with LowHp cows.

In our study, plasma aliquots were handled equally among groups of cows and were immediately placed in thermo-conductive modules after sampling for rapid sample cooling. The samples in this study were stored under −80°C, and never submitted to a freeze-thaw cycle before LC-MS/MS analysis. If stability issues occurred during storage, all samples were submitted to a nondifferential bias, and the percentage of error was assumed to be approximately equal between the groups being compared. If true, our study could have failed to detect small differences in oxylipins across groups of cows and could only identify major differences. No comprehensive data exist delineating best sampling management practices for bovine plasma oxylipin measurements (e.g., types of anticoagulants used, the effect of sample storage time and temperature, and the addition of oxidative inhibitors). Therefore, studies evaluating bovine plasma oxylipin stability under long-term storage are needed.

Our study demonstrates the relationship between specific pro- and anti-inflammatory oxylipins and the degree of systemic inflammation suffered by transition dairy cows. Previous studies have investigated the relationship between oxylipin profiles and dairy cattle health outcomes (Raphael et al., 2014; Putman et al., 2022). However, research using larger sample sizes is needed to reinforce the importance of our results and best define the in vitro and in vivo functions of the oxylipins identified for dairy cows.
